# The visual outcomes and complications of manual small incision cataract surgery and phacoemulsification: long term results

**DOI:** 10.22336/rjo.2021.7

**Published:** 2021

**Authors:** Kamonporn Nampradit, Pipat Kongsap

**Affiliations:** * Department of Ophthalmology, Prapokklao Hospital, Thailand

**Keywords:** manual small incision cataract surgery, phacoemulsification, long term complications, visual acuity, pseudophakic bullous keratopathy, visual acuity

## Abstract

**Objective:** To compare visual outcomes and complications between manual small incision cataract surgery (MSICS) and phacoemulsification.

**Methods:** A retrospective study was conducted in the tertiary care center. A total of 1281 cases underwent manual small incision cataract surgery and phacoemulsification from January 2014 to December 2016. The postoperative best corrected visual acuity (BCVA) along with the rates of complications were compared between both groups.

**Results:** Five hundred and twenty-one patients (40.67%) and 760 patients (59.33%) were subjected by staff members and residents, respectively. Altogether, 689 cases (53.79%) were subjected to MSICS technique and 592 cases (46.21%) to phacoemulsification. The MSICS group had significantly harder cataract (cataract grading ≥ 4+ :31.64% vs. 7.77%; p<0.001). One month postoperatively, good visual outcome (BCVA ≥ 6 /18) in the phacoemulsification group was higher than that in the MSICS group (86.33% vs. 72.12%, p<0.001). The risk factor for poor outcome (post-operative BCVA < 6 /60 in both groups) was the presence of associated ocular pathologies. The intraoperative and perioperative complications rates were higher in the MSICS group (16.55% vs. 6.6%, p<0.001). The most common complications were hyphema (4.35%), posterior capsule ruptures (4.21%), and prolapsed iris (3.05%). Long-term postoperative complication rates were higher in the phacoemulsification group (9.29% vs. 21.28%, p<0.001). The most common complication was posterior capsule opacity (8.71% vs. 20.44%, p<0.001). Pseudophakic bullous keratopathy (PBK) was similar in both groups (0.29% vs. 0.17%, p=1.00).

**Conclusion:** The number of patients who had experienced good visual outcomes was higher in the phacoemulsification group. However, for both groups, no significant differences were found on the long-term complication rate.

## Introduction

Cataracts, which are the number one cause of blindness, can be treated by surgery [**1**]. The most common cause of cataracts is due to the changes caused by the aging of the lens [**2**]. At present, there are a variety of common cataract surgery methods as such as the following: 1) Phacoemulsification (PE) - the cataract is removed by an ultrasonic device (Phaco machine), which is considered to be the most popular surgery that is being currently used due to the small incision of approximately 3 millimeters in size, which results in a speedy recovery from postoperative wound despite the high cost; 2) Extracapsular cataract extraction (ECCE) is a surgery requiring the creation of a large surgical wound (approximately 8-10 millimeters in size) and multiple stitches, which lead to a long time for recovery and more astigmatism after surgery; and 3) Manual small incision cataract surgery (MSICS), a variety of surgical techniques currently available [**3**-**10**]. For instance, Prapokklao Hospital in Thailand has performed cataract surgery using manual small incision cataract surgery (MSICS) and has developed a surgical procedure that can be performed by using various techniques, such as Modified Blumenthal technique, Nylon loop technique, Kongsap’s technique, and the Ruit’s technique [**11**,**12**]. The Modified Blumenthal technique has been used for small incision cataract surgery for patients at Prapokklao Hospital for over 20 years. However, at present, there is no comparative study of the post-operative visual outcomes and long-term complications from cataract surgery by manual small incision cataract surgery (MSICS) and by phacoemulsification in Thailand. 

In general, cataract surgery is safely controlled by The World Health Organization (WHO) and is carried out through a set of standardized surgical procedures, which are safe for patients with a good level of vision, by determining a good result to be at least 90 percent and a poor result to be not more than 5% [**13**]. Phacoemulsification is a better method when compared with other methods [**4**,**14**,**15**]. However, in cases in which there are very hard or milky white cataracts, it has been found that manual small incision cataract surgery provides better and safer surgical results even though results emerging from the long-term surgery, including its complications over 1 year, have not yet been reported [**16**,**17**].

The objective of this study was to compare the level of vision of post-operative visual outcome and the long-term complications with reference to manual small incision cataract surgery (MSICS) and cataract surgery using phacoemulsification.

## Materials and Methods

This research was a retrospective comparative study and its ethical approval was reviewed by the Human Ethics Committee of Prapokklao Hospital. The data was collected from the medical records of patients, who underwent cataract surgery at Prapokklao Hospital in Chanthaburi from January 2014 to December 2016. The data in the medical records encompassed the sex, age, systemic diseases, and ocular diseases with regards to the patient. Each patient underwent an eye examination with the aid of a slit lamp under the supervision of an ophthalmologist. The examination included the measurement and assessment of visual acuity, intraocular pressure, nucleus hardness/ lens opacities [**18**], fundus examination, keratometry prior to surgery, the date and time of the surgery, laterality of the eye that underwent the surgery, the surgical method, the surgeons (Staff Member or Resident), any complications that occurred during surgery and after surgery, the type of artificial intraocular lens inserted in the eye of the patient, post-operative visual acuity, and the follow-up appointment. This data was collected on the research form.

The underlying systemic diseases were diabetic mellitus (DM), hypertension (HT), dyslipidemia (DLP), cerebrovascular accident (CVA), epilepsy, ischemic heart disease (IHD), cardiomyopathy, atrial fibrillation (AF), chronic obstructive pulmonary disease (COPD), chronic kidney disease (CKD), gout, psoriasis, cirrhosis, anemia, benign prostatic hyperplasia (BPH), rheumatoid arthritis (RA), Parkinson’s disease, anxiety, malignancies, and others.

The underlying ocular diseases were glaucoma, corneal scars, age-related macular degeneration (AMD), proliferative diabetic retinopathy (PDR), non-proliferative diabetic retinopathy (NPDR), diabetic macular edema (DME), retinitis pigmentosa (RP), macular holes (MH), epiretinal membrane (ERM), Vogt-Koyanagi-Harada (VKH), macular scars, optic disc atrophy, chronic retinal detachment (RD), branch retinal vein occlusion (BRVO), central retinal vein occlusion (CRVO), vitreous hemorrhage (VH), zonule dialysis, amblyopia, uveitis, and refractive errors.

In the WHO recommendations on the outcome of cataract surgery, the visual acuity values were the following: good visual acuity (6/ 6-6/ 18), borderline visual acuity (< 6 /18-6 /60), and poor visual acuity (< 6/ 60) [**13**].

The classification to determine the hardness of the cataract was carried out in accordance with the lens opacities classification system III [**18**] as it follows: 1) by Nuclear sclerosis ≤ 3+; 2) Cortical and PSC were considered as “soft cataracts”; 3) Nuclear sclerosis ≥ 4+, and 4) mature and hyper-mature cataracts were considered as “hard cataracts”.

The following surgical cataract methods were used: 1) MSICS including Modified Blumenthal technique, which was most used, the K-MPF technique, the Nylon loop technique, and the Ruit technique; and 2) cataract surgery by Phacoemulsification.

The surgeons consisted of: 1) staff members and 2) 3rd year ophthalmology residents, who were supervised by the staff members.

Intraoperative /perioperative complications consisted of capsule rupture, drop nucleus, hyphema, iridodialysis, prolapsed iris, suprachoroidal hemorrhage, Descemet membrane stripping, zonule dialysis, tears in the anterior capsule, corneal edema, endophthalmitis, IOL malposition/ dislocation, and secondary glaucoma.

Long term complications included cystoid macular edema (CME), pseudophakic bullous keratopathy (PBK), retinal detachment (RD), posterior capsule opacity (PCO), and IOL malposition/ dislocation.

The patients were examined at follow-up appointments at the following intervals: 1 day, 1 week, 3 months, 6 months, 1 year after the surgery, and once a year thereafter.

The inclusion criteria were the following: first, the cataract patients underwent manual small cataract surgery (MSICS) and phacoemulsification, and second, the patients who had follow-up examinations for at least 1 year.

The exclusion criteria were the following: 1) cataract patients, who underwent other surgical methods, such as ICCE, ECCE; and 2) cataract patients whose follow-up examinations were lost.

**Sample size calculation:** The sample size was calculated by the software based on 50 cases from the pilot study. The visual acuity ratio of the MSICS and the Phacoemulsification operations was 90.4%: 95.5%. The Power of the test = 0.9, while the Significant level (alpha) = 0.5. A two-sample comparison of the proportion was used to find the estimated required sample size (approximately 566 cases per group and an additional 10% in case of incomplete data), which resulted in a total of 1,200 cases. 

**Research Statistics:** A software package was used to carry out the statistical calculations. For variables with normal distribution, numeric data was compared between various groups using Exact probability, the Independent t-test, and the Chi-square test. Meanwhile, the Wilcoxon signed rank test was used for variables with abnormal distribution. In addition, a P value of <0.05 was significant.

## Results

After a careful review of the medical records of the outpatient cataract patients, who underwent cataract surgery from January 2014 to December 2016, it was observed that a total of 1281 patients were eligible for the study, consisting of 743 female patients and 538 male patients within the range of 18-92 years of age (the group mean age of the MSICS patients was found to be 70.44 years, while the group mean age of the Phacoemulsification patients was found to be 69.23 years). 689 patients underwent manual small incision cataract surgery (MSICS), while 592 patients underwent cataract surgery via Phacoemulsification. Lens hardness at NS ≤ 3 + was found in 471 cases (68.36%) of MSICS and in 543 cases (92.23%) of Phacoemulsification. In contrast, lens hardness at NS ≥ 4 + was found in 218 cases of MSICS (31.64%) and in 46 cases (7.77%) of Phacoemulsification.

The preoperative visual acuity (VA pre-op) < 6/ 60 for MSICS was 447 cases (64.88%) and for Phacoemulsification was 214 cases (36.15%), while the VA pre-op < 6/ 18-6/ 60 for MSICS was 239 cases (34.69%) and for Phacoemulsification was 364 cases (61.49%).

Ocular diseases were found in 85 cases (12.34%) of MSICS and in 90 cases (15.2%) of Phacoemulsification.

The average operation time in MSICS surgery was 42.44 minutes, while the average time spent in Phacoemulsification surgery was 27.5 minutes.

In the MSICS group, 403 patients (58.49%) were performed by residents, while only 118 patients (19.93%) were performed by residents in the Phacoemulsification group.

PMMA was used for the MSICS group in 605 cases (87.81%). For the phacoemulsification group, the artificial lenses were Hydrophilic acrylic in 447 cases (75.51%), and PMMA in 133 cases (22.47%). The average times for the follow-up examination were 88.4 weeks and 104.5 weeks for the MSICS group and the phacoemulsification group, respectively (**Table 1**).

**Tabel 1:** Demographic and clinical characteristics

Demographic and clinical characteristics	MSICS (n= 689)		Phacoemulsification (n= 592)		p-value
	n	%	n	%	
Gender					
Male	288	41.8	250	42.23	0.0342
Female	401	58.2	342	57.77	
Age (year)					
Mean ± sd	70.44	10.98	69.23	9.23	0.0336
Systemic disease					
no	197	28.59	179	30.24	0.519
yes	492	71.41	413	69.76	
Ocular disease					
no	604	87.66	502	84.80	0.136
yes	85	12.34	90	15.20	
Laterality					
R	356	51.74	319	54.37	0.407
L	333	48.26	273	45.63	
Nuclear opacity					
NS ≤ 3+	471	68.36	546	92.23	<0.001
NS ≥ 4+	218	31.64	46	7.77	
Pre-op VA					
6/ 6-6/ 18	3	0.43	14	2.36	<0.001
< 6/ 18 - 6/ 60	239	34.69	364	61.49	
< 6/ 60	447	64.88	214	36.15	
Duration of Surgery (min)					
Mean+-SD	42.44	17.39	27.5	11.3	<0.001
Surgeon					
Staff	286	41.51	474	80.07	
Resident	403	58.49	118	19.93	
Intra ocular lens type					
Polymethyl methacrylate (PMMA)	605	87.81	133	22.47	<0.001
Hydrophilic acrylic	80	11.61	447	75.51	
Hydrophobic acrylic	4	0.58	12	2.02	
Follow-up (weeks)	88.42	57.34	104.48	67.4	<0.001

Visual acuity of post-operative cataract patients undergoing MSICS was the following: 1) at 1 month of VA 6/ 6-6/ 18 445 cases (72.12%), VA < 6/ 18-6/ 60 146 cases (23.66%); 2) at 3 months VA 6/ 6-6/ 18 322 cases (76.12%), VA < 6/ 18-6/ 60 88 cases (20.81%); and 3) the VA at the last follow-up appointment (> 1 year) VA 6/ 6-6/ 18 507 cases (73.58%), VA < 6/ 18-6/ 60 151 cases (21.92%) (**Table 2**).

The visual acuity of the post-operative cataract patients undergoing Phacoemulsification was the following: 1) at 1 month of VA 6/ 6-6/ 18 417 cases (86.33%), VA < 6/ 18-6/ 60 55 cases (11.39%); 2) at 3 months, VA 6/ 6-6/ 18 313 cases (85.99%), VA < 6/ 18-6/ 60 39 cases (10.71%), and 3) the VA at the last follow-up appointment (> 1 year): VA 6/ 6-6/ 18 485 cases (81.93%), VA < 6/ 18-6/ 60 79 cases (13.34%) (**Table 2**).

**Tabel 2:** The clinical outcomes

Visual acuity	MSICS		Phacoemulsification		p-value
	N	%	n	%	
Post op VA 1 Mo					
6/ 6 - 6/ 18	445	72.12	417	86.33	<0.001
< 6/ 18 - 6/ 60	146	23.66	55	11.39	
< 6/ 60	26	4.22	11	2.28	
Post op VA 3 Mo					
6/ 6 - 6/ 18	322	76.12	313	85.99	<0.001
< 6/ 18 - 6/ 60	88	20.81	39	10.71	
< 6/ 60	13	3.07	12	3.3	
Post op VA Last F/ U(>1yr)					
6/ 6 - 6/ 18	507	73.58	485	81.93	<0.001
< 6/ 18 - 6/ 60	151	21.92	79	13.34	
< 6/ 60	31	4.5	28	4.73	

Regarding MSICS, it was found that there were 114 cases of intraoperative/ perioperative complications, consisting of 30 cases of hyphema (4.35%), 29 cases of ruptured posterior capsules (4.21%), 22 cases of vitreous loss (3.19%), 21 cases of prolapsed iris (3.05%), 15 cases of corneal edema (2.18%), 8 cases of Descemet stripping (1.16%), and 5 cases of iridodialysis (0.73%), etc. (**Table 3**).

With respect to the cataract surgery performed through the method of Phacoemulsification, there were 40 cases of intraoperative/ perioperative complications, including 18 cases of ruptured posterior capsules (3.05%), 6 cases of vitreous loss (1.02%), 8 cases of corneal edema (1.35%), 6 cases of Descemet stripping (1.02%), and 3 cases of tears to the anterior capsule (0.51%), etc. (**Table 3**).

**Tabel 3:** The intraoperative/ perioperative complications

Intraoperative/ Perioperative Complications	MSICS (n=689)		Phacoemulsification (n=592)		p-value
	N	%	n	%	
No	575	83.45	552	93.4	<0.001
Yes	114	16.55	40	6.6	
Posterior capsule tear (No Vit loss)	7	1.02	12	2.03	
Posterior capsule tear (Vit loss)	22	3.19	6	1.02	
Tear anterior capsule	1	0.15	3	0.51	
Iris prolapse	21	3.05	2	0.34	
Iridodialysis	5	0.73	0	0.0	
Zonule dialysis	4	0.58	2	0.34	
Descemet’s stripping	8	1.16	6	1.02	
Drop nucleus	0	0.0	0	0.0	
Suprachoroidal hemorrhage	1	0.15	0	0.0	
Hyphema	30	4.35	1	0.17	
Corneal edema	15	2.18	8	1.35	

Regarding the long-term postoperative complications, 64 cases of complications (9.29%) were found in the MSICS group, comprising 60 cases of posterior capsule opacity (8.71%), 2 cases of pseudophakic bullous keratopathy (0.29%), 1 case of IOL malposition (0.15%), and 1 case of cystoid macular edema (0.15%). However, no cases of endophthalmitis or retinal detachment were found (**Table 4**).

Meanwhile, it was found that in the phacoemulsification group, there were 126 cases of long-term complications (21.28%), consisting of 121 cases of posterior capsule opacity (20.44%), 2 cases of IOL malposition (0.34%), 1 case of pseudophakic bullous keratopathy (0.17%), 1 case of cystoid macular edema (0.17%), and 1 case of retinal detachment (0.17%). Furthermore, no cases of endophthalmitis were found (**Table 4**). 

**Tabel 4:** The long-term post-operative complications

Post-operative Complications	MSICS (n=689)		Phacoemulsification (n=592)		p-value
	n	%	n	%	
No	625	90.71	466	78.72	<0.001
Yes	64	9.29	126	21.28	
Posterior capsule opacity	60	8.71	121	20.44	
Endophthalmitis	0	0.0	0	0.0	
IOL malposition	1	0.15	2	0.34	
Cystoid macular edema	1	0.15	1	0.17	
Retinal detachment	0	0.0	1	0.17	
Pseudophakic bullous keratopathy	2	0.29	1	0.17	

## Discussion

With respect to the manual small incision cataract surgery (MSICS), good visual outcomes (VA 6/ 6 - 6/ 18) were found as compared to the WHO Visual acuity after surgery at 1 month, 3 months, and at the last follow up (> 1 year) because it was still found to be below the threshold (based on the WHO criteria set to be over 90%). On the contrary, poor visual outcomes (VA < 6/ 60) in this study outperformed the criteria (criteria set to be less than 5%). The cause of poor visual acuity after surgery was likely due to the underlying ocular diseases of the patients, such as AMDs, glaucoma, PDR, DME, MH, ERM, macular scars, optic disc atrophy, RP, RD, BRVO, chronic uveitis, amblyopia, corneal scars, or refractive errors.

Regarding intraoperative/ perioperative complications, the MSICS group had more complications than the Phacoemulsification group (16.5% and 6.6%, respectively), which was consistent with the results from the studies of Rohit C Khanna et al. and Aravind Hairpriya et al., who typically found that the complications were hyphema (4.35%), posterior capsule rupture (3.34%), and iris prolapse (3.05%) [**19**,**20**]. Moreover, corneal edema (2.18%) was found one day after the surgery. This was because surgery was mostly conducted at the scleral tunnel and it was likely that trauma to the iris had occurred during lens subluxation and lens delivery. In the MSICS group, the surgeons team consisted of 58.49% residents, who had less experience and less expertise than the staff members [**21**]. This was likely to have caused more complications than in the Phacoemulsification group because most of the surgeons were staff members (80.07%).

Considering the late postoperative complications, complications from phacoemulsification were found more often than with surgery using MSICS, especially posterior capsule opacity: PCO (Phacoemulsification 20.44%, MSICS 8.71%). According to the subgroup analysis, it was found that the type of artificial lenses that were used the most was Hydrophilic acrylic (75.51%) in the Phacoemulsification group. Therefore, the PCO was greater than in the MSICS surgery in which Polymethyl methacrylate (PMMA) (87.81%) was implanted, while only 11.61% were implanted with Hydrophilic acrylic intraocular lenses.

Pseudophakic bullous keratopathy (PBK), which is a late serious postoperative complication is also another indicator of safety of each of the operative methods. In this study, patients were monitored for over 1 year (on average about 80 weeks after MSICS surgery), and it was found that there were 2 cases of PBK (0.29%) compared to 1 case of Phacoemulsification (0.17%). No significant difference was discovered, and it was found to be less than the incidence in cataract surgery (1-2%) [**22**].

Post-operative endophthalmitis was not found in this study, and the incidence was lower than in the United States (0.04%) [**23**].

**Limitations in this research study**

1) Since this was a retrospective study, information about complications was likely to be collected less than the real numbers.

2) Patient characteristics in both groups of patients were different from the beginning, which caused discrepancies between both groups of patients.

3) Regarding the surgeries, residents performed 58.49% of the MSICS and 19.93% of the phacoemulsification of all patients, which resulted in a longer duration of surgery, as well as in more complications in the MSICS group than usual.

## Conclusions

Regarding the post-operative visual acuity results, the cataract patients, who underwent Phacoemulsification, had significantly better visibility than those receiving MSICS at 1 month, 3 months, and more than 1 year after the surgery. However, late postoperative complications were found more frequently in the Phacoemulsification group as compared to the MSICS group. Most of the complications dealt with the posterior capsule opacity (PCO) since hydrophilic acrylic lenses had mostly been implanted in the Phacoemulsification group. Meanwhile, no differences between the MSICS group and the Phacoemulsification group were found with respect to the long-term complication rate.

**Conflict of Interest**

Authors state no conflict of interest.

**Informed Consent and Human and Animal Rights statements**

Informed consent has been obtained from all individuals included in this study.

**Authorization for the use of human subjects**

Ethical approval: The research related to human use complies with all the relevant national regulations, institutional policies, is in accordance with the tenets of the Helsinki Declaration, and has been approved by the Human Ethics Committee of Prapokklao Hospital.

**Acknowledgements**

The article has not been presented in any meeting. 

**Sources of Funding**

This study was funded in-part by the Prapokklao Hospital research and development fund.

**Disclosures**

None.
